# Analysis into the viability of pea gravel as a diffusing material for biostimulation systems in petroleum hydrocarbon‐contaminated soils

**DOI:** 10.1002/jeq2.70114

**Published:** 2025-12-17

**Authors:** Aimée D. Schryer, Alejandro Alvarez Ruiz, Kris Bradshaw, Steven D. Siciliano

**Affiliations:** ^1^ Department of Soil Science, College of Agriculture and Bioresources University of Saskatchewan Saskatoon Saskatchewan Canada; ^2^ Department of Plant and Environmental Sciences University of Copenhagen Frederiksberg Denmark; ^3^ Federated Cooperatives Limited Saskatoon Saskatchewan Canada

## Abstract

Injecting biostimulatory solutions around underground storage tanks (UGSTs) promotes petroleum hydrocarbon (PHC) bioremediation without the use of intrusive infrastructure. Yet, it is unclear how the interaction between porous bedding material (i.e., pea gravel) and biostimulatory solutions impacts PHC biodegradation rates. Consequently, we assessed whether pea gravel can act as an inert diffusion material that facilitates PHC bioremediation within UGST beds. First, we evaluated how biostimulatory solution elution through pea gravel (i.e., weathered) altered nutrient composition and benzene degradation rates. Suspected microbial activity decreased the proportion (i.e., nutrient concentration after pea gravel elution divided by initial biostimulatory solution concentration) of ammonium (0.45–0.84) and citrate (0.0–0.25) in effluents. In comparison, the proportion of anaerobic electron acceptors sulfate (0.91–1.08) and nitrate (0.78–1.0) were largely unaffected by pea gravel sorption. After 28 days, the weathered biostimulatory solution degraded 25 ± 0.50% of added benzene at rates similar (0.010 ± 0.002 days^−1^) to fresh biostimulatory solution (0.008 ± 0.001 days^−1^). Next, using positron emission tomography (PET) with [^18^F]‐fluorodeoxyglucose and [^18^F]‐fluoride, we evaluated the risks of biofilm fouling to gravel beds. In agreement with the reduced nutrient concentration, PET results show that the biostimulatory solution promoted microbial growth on the surface of the pea gravel. Yet, as the pore space occupied by bacteria remained low (between 8.8% and 13%) following biostimulatory solution elution, biofilm formation is not a concern. Therefore, pea gravel environments in tank nest areas can support PHC biostimulatory solution delivery with minimal consequences.

## Core Ideas

- The impact of underground storage tank nest fill (gravel) on petroleum‐hydrocarbon bioremediation rates is unknown.
- Biostimulatory solution effluent from pea gravel elution promoted the largest benzene biodegradation rates.
- Pea gravel promotes little microorganism proliferation following biostimulatory solution elution.

## Plain Language Summary

Petroleum hydrocarbons (PHCs), such as gasoline and diesel, are often stored in underground storage tanks (UGSTs). When an UGST leaks products into nearby soil, it poses a risk to both humans and the environment. A common method for cleaning up PHC spills around tanks is to harness the power of soil microorganisms by providing a nutrient‐rich start‐up solution (biostimulatory solution). Our experiment aimed to investigate how pea gravel, commonly used to pack UGST, affects the fate and efficiency of biostimulatory solutions. To resolve this question, pea gravel cores were used to simulate tank bed conditions. Results showed that while some components (ammonium and citrate) were taken up by microbes, the loss of these nutrients did not negatively affect PHC breakdown in soil samples. At the same time, moving the biostimulant through pea gravel did not cause an excessive growth of microorganisms. We concluded that pea gravel will not inhibit and may even improve PHC degradation.

**Abbreviations**
[^18^F]FDG: [^18^F]fluorodeoxyglucose
FDG: fluorodeoxyglucose
G6‐PO_4_
: glucose‐6‐phosphate
GC: gas chromatograph
IC: ion chromatography
ISO: International Organization for Standardization
LB: lysogeny broth
MSA: methanesulfonic acid
PET: positron emission tomography
PHC: petroleum hydrocarbon
SPME: solid phase micro‐extraction
TPP: tripolyphosphate
UGST: underground storage tank

## 1 INTRODUCTION

While refined crude oil or crude oil‐based products (e.g., gasoline and diesel products) support numerous sectors of modern society, improper discharges create environmental and human health concerns (Canadian Council of Ministers of the Environment [CCME], 2008). Any anthropogenic incidents, such as accidental releases and discharges into the environment from underground fuel storage facilities, negatively modify soil structure and function (Fei‐Baffoe et al., 2024; Li et al., 2023). One remediation approach is bioremediation, which uses the natural degradative ability of microorganisms to reduce petroleum hydrocarbon (PHC) into less toxic by‐products (Lai et al., 2009; Mamet et al., 2021). As natural pollutant removal rates are low, largely due to limited nutrient bioavailability, environmental factors, and the activity of microorganisms, a biostimulatory solution provides nutrients and electron acceptors to correct nutrient ratio imbalances (Júlio et al., 2018; Siciliano et al., 2016). However, biostimulation degradation rates depend on biological, physical, and chemical factors (Mohajeri et al., 2017), including substrate particle size within the treatment area.

**FIGURE 1:**
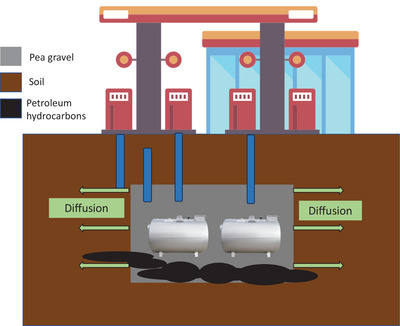
Graphical representation of the underground storage tank gravel nesting design commonly employed in Western Canada. Petroleum hydrocarbon (PHC) contamination (black circles) occurs around the tank and pipelines and will accumulate in the pea gravel. Mass flux and diffusive processes cause PHC to move from the gravel to the finer particles surrounding the underground storage tank nest.

Spills during vehicle refueling and leaks from underground storage tanks (UGSTs) and associated pipelines cause subsurface contamination to the underlying tank bed (Rosales et al., 2014). These areas comprise highly porous bedding material, like sand or pea gravel (>2 mm), replacing the excavated soil around the tank (Figure 1; Government of Saskatchewan, 2020; Lin et al., 2023). The storage tank areas require special attention as the nest will accumulate and promote lateral movements of PHC at increasing distances from the tank area (Gao et al., 2024). The high permeability of gravel within the tank bed could be used as an injection medium, possibly reducing the implementation and operation costs compared with traditional biostimulation injection systems. However, the potential of pea gravel to promote biostimulatory solution dispersion and diffusion has not been studied.

Due to the low ion sorption capacity of gravel, biostimulatory solution flow and nutrient availability may increase for PHC microbial degradation compared to other backfill materials, including soil. Zhu et al. (2011) evaluated the kinetic sorption of ammonium (NH_4_) to gravel using batch experiments and kinetic sorption isotherms. The authors found that the maximum NH_4_ sorption capacity of gravel was 769 mg kg^−1^, while the cation exchange capacity was 10 ± 0.2 cmol_c_ kg^−1^, the second lowest among the studied materials. Gravel also showed low exchangeable Ca, Mg, and active Fe, Mn, K, Ca, Cu, and Zn values, indicating poor ion retention (Zhu et al., 2011).

As biofilm formation from the eutrophic growth of microorganisms causes physical operation issues such as plugging and the reduction of injection system flow rates, practitioners must prevent the development of an interfering microscale ecosystem (i.e., biofouling; Sanders & Sturman, 2005). Biofilm production follows a predictable route. Bacteria and other microorganisms adhere to surfaces as a survival mechanism (Kochkodan & Hilal, 2015). As microorganisms accumulate, an agglomeration of extracellular materials onto or within the pores of a surface occurs, followed by biofilm formation of extracellular polymeric substances (Al‐Juboori & Yusaf, 2012; Kim et al., 2019; Yang et al., 2020). Biofouling of the surface of synthetic polymer membranes used in water treatment applications is a commonly encountered problem that can dramatically diminish the treatment process efficiency and cost‐effectiveness (Kochkodan & Hilal, 2015). Microbes on surfaces lead to the corrosion and biodeterioration of numerous materials (Sanders & Sturman, 2005). Finally, live and dead bacteria can clog preferential soil flow paths, altering the transport routes for biostimulatory solution and PHC compounds (Mamet et al., 2021).

Investigations into biofouling risks require methodologies capable of identifying microbial activity. Positron emission tomography (PET) is a nondestructive imaging technique for measuring the biological functioning of living organisms (Garbout et al., 2012). PET enables mapping tracer uptake, utilization, and excretion in living organisms marked with positron‐emitting radionuclides in the same specimen over time (Ariño‐Estrada et al., 2019). As glucose is a primary source of energy, using fluorodeoxyglucose (FDG) (which assumes parallel glucose‐6‐phosphate [G6‐PO_4_] metabolism) allows for heterotrophic microbial activity (Roth et al., 2024; Schmidt et al., 2020). Unlike G6‐PO_4_, FDG metabolism halts at fluorodeoxyglucose‐6‐phosphate (FDG‐6‐PO_4_) in the glycolysis pathway, causing accumulation within cells (Schmidt et al., 2020). Therefore, adding [^18^F]fluorodeoxyglucose ([^18^F]FDG) causes the accumulation of [^18^F]fluorodeoxyglucose‐6‐phosphate ([^18^F]FDG‐6‐PO_4_), allowing the visualization of active microbial communities (Galieni et al., 2021). Previous work combined PET with [^18^F]FDG to improve PHC bioremediation efforts within intact cold, northern region soil cores (Mamet et al., 2021). By imaging pore volumes and bacterial activity, the authors identified the dominant taxa responsible for PHC and identified the biostimulatory solution components that optimized phosphate bioavailability.

In this study, we aimed to investigate whether pea gravel, a material commonly used as tank bed fill material, could alter PHC bioremediation through biotic (i.e., microorganisms) and abiotic (e.g., sorption) processes. We hypothesized that (1) injecting biostimulatory solutions through a gravel backfill will not affect the biostimulatory solution chemical composition or nutrient bioavailability for PHC‐degrading microorganisms, (2) biostimulatory solution passed through gravel will promote PHC bioremediation in gravel and soil matrices, and (3) biofouling will not occur from saturated gravel. Three experiments were conducted to resolve these hypotheses. The first trial constructed cores to examine the sorption capacity of gravel in contact with a biostimulatory solution via breakthrough curves. A second trial evaluated whether the biostimulatory solution following pea gravel contact promoted microbial degradation in benzene‐doped anaerobic soil microcosms. Finally, two ^18^F radiotracers (F^−^(aq)/FDG) were used to observe biofouling risks by pumping the radioisotopes through gravel cores in contact with a biostimulatory solution to identify bacterial activity and volumes. By creating these trials, we evaluated whether gravel‐filled tank beds promote bioremediation in the epicenter of PHC contamination, thereby minimizing area disturbances during bioremediation.

**Core Ideas**

- The impact of underground storage tank nest fill (gravel) on petroleum‐hydrocarbon bioremediation rates is unknown.
- Biostimulatory solution effluent from pea gravel elution promoted the largest benzene biodegradation rates.
- Pea gravel promotes little microorganism proliferation following biostimulatory solution elution.

## 2 MATERIALS AND METHODS

### 2.1 IRC biostimulatory solution composition

Previous work during long‐term bioremediation trials in SK, Canada, established a bioremediation recipe termed the IRC (Industrial Research Chair) biostimulatory solution (Bulmer et al., 2018; Chen et al., 2017; Hamilton et al., 2018; Huang et al., 2019; Siciliano et al., 2016). The recipe consists of 0.24 mM HNO_3_ (3.4 mg L^−1^ N), 0.24 mM Fe (III), NH_4_‐citrate (13 mg L^−1^ Fe [III]), and 0.1 mM tripolyphosphate (TPP; 3.1 mg L^−1^ P). The nutrient mix BioLodestone (Environmental Material Science) with added sulfate (MgSO_4_) was added to supplement the IRC biostimulatory solution.

Columns were filled with approximately 186 g of pea gravel, occupying a volume of 113 cm^3^. Ion exchange resin (Amberlite MB20, 428736, Sigma‐Aldrich) and glass beads (Cole‐Parmer) acted as high and null sorption controls. Two treatments (IRC solution and Milli‐Q [Millipore Sigma, Model Direct 16]) were pumped through columns (three experimental units for the pea gravel columns and one experimental unit for the glass bead and resin controls), with initial ion concentrations provided in Table 1. Treatments were injected through the columns using a peristaltic pump (Model IPC ISM939D, ISMATEC) and precision pump tubing (96410‐14, Masterflex) at a rate of 0.1 mL min^−1^. The columns included an outlet tube at the bottom for effluent sampling and draining. A portion of the effluent (3 mL) at days 1, 3, 5, 7, 9, 12, 15, 18, 21, 24, 27, and 30 was collected for solution cation and anion concentration analysis.

**TABLE 1:** Mean ion concentration (*n* = 10) ± standard deviation of the IRC solution and Milli‐Q water. The concentration within each treatment was measured using an integrated ion chromatograph system.

Ion	Treatment	
	IRC solution (mg L^−1^)	Milli‐Q water (mg L^−1^)
Ammonium	7.5 ± 0.4	<LLOQ^a^
Calcium	0.7 ± 0.1	<LLOQ
Citrate	36 ± 5.2	<LLOQ
Magnesium	17 ± 1.1	<LLOQ
Nitrate	16 ± 0.5	<LLOQ
Sodium	12 ± 0.9	<LLOQ
Sulfate	62 ± 2.8	<LLOQ
Phosphate	16 ± 0.4	<LLOQ

Abbreviation: LLOQ, lower limit of quantification.

^a^ Values below the lower limit of quantification.

Anion concentration (SO_4_, NO_3_, PO_4_, and citrate) in both fresh and effluent amendment solutions was analyzed using an integrated ion chromatography (IC) system (DIONEX ICS‐2000, Thermo Fisher Scientific), which comprises an eluent generator, an isocratic pump, and a conductivity detector. The system is attached to the DIONEX AS‐DV (Thermo Scientific) sampler. The effluent generation was fulfilled using an eluent generator cartridge III potassium hydroxide (KOH) (074532, Thermo Scientific).

Anion quantification started by injecting 25 µL of the sample into a 25‐µL injection loop. The loop is connected to a column (Dionex IonPac AS18 4 mm × 150 mm; Thermo Scientific), with the guard column (Dionex IonPac AG18 4 mm × 50 mm; Thermo Scientific) and a suppressor (AERS 500e 4 mm, 302661; Thermo Fisher Scientific). The flow rate was constant at 1.0 mL min^−1^ and a 30°C column temperature. Detection was completed using suppressed conductivity. The KOH eluent followed a multi‐step concentration gradient. At the onset of sample introduction, KOH concentration was 32.0 mM and held for 5 min. After 5 min, KOH concentrations ramped to 60.0 nM over 2.5 min (end at 7.5 min) and held for 30 s (end at 8.0 min). For 0.1 min, concentrations are pushed to 100 mM (end at 8.1 min) and held for 3.9 min (end at 12 min). The KOH concentration decreased to 32 mM rapidly (end at 12.1 min) before holding for 2.9 min (end at 15.0 min).

The same IC system completed cation analysis (Na, Ca, Mg, and NH_4_). The eluent generation was fulfilled using an eluent generator cartridge III methanesulfonic acid (MSA) (074535, Thermo Scientific). Briefly, 25 µL of the sample was injected into a 25‐mm injection loop to a column (Dionex IonPac CS12A 4 mm × 250 mm; Thermo Scientific), with a guard column (Dionex IonPac CG12A 4 mm × 50 mm; Thermo Scientific) and a suppressor (CDRS 600 4 mm, 088666; Thermo Scientific). The flow rate was 1.5 mL min^−1^ throughout the sample analysis with a 30°C column temperature. Detection was completed using suppressed conductivity. The MSA eluent concentration varied by run time (total of 17 min): Briefly, the run starts at 5.0 mM before gradually increasing to 20.0 mM for 5.9 min (end at 6.0 min). The MSA eluent concentration was kept constant for 6 min (end at 12.0 min) before decreasing to 5.0 mM for 5 min (end at 17.0 min).

Chromeleon (version 7.2.9, Thermo Scientific) processed anions and cation analysis. The retention time‐identified species were quantified using an external calibration curve using a mixture of diluted compounds from a commercial stock solution. For anions, the species of interest included F (77365, Sigma), Cl (39883, Sigma), SO_4_ (90071, Sigma), NO_3_ (74246, Sigma), PO_4_ (38364, Sigma), and citrate (96068, Sigma). For cations, the commercial stock solutions included Na (43492, Sigma), NH_4_ (59755, Sigma), K (53337, Sigma), Mg (89441, Sigma), and Ca (39865, Sigma).

### 2.2 The efficiency of biostimulation solution effluents for benzene degradation after flowing through gravel columns

Anaerobic microcosms assessed the efficiency of the biostimulatory solution effluents (and controls) from Section 2.2 to promote benzene degradation, with the schematic presented in Figure S1. Five treatments were used for this experiment with three replicates: (1) freshly prepared IRC solution, (2) Milli‐Q water, (3) IRC solution effluent from the column trial (Section 2.2), (4) Milli‐Q effluent from the column trial, and (5) abiotic controls. Experimental units were created in 125‐mL serum bottles (223748, Wheaton) containing a soil sample collected from an active remediation site in SK, Canada (51.142581°, −105.443276°). The soil pH is 7.9 (5 g soil to 10 mL Milli‐Q water; Food and Agriculture Organization [FAO], 2021), with total alkalinity at 62 mg L^−1^ (International Organization for Standardization [ISO] 10694; International Organization for Standardization [ISO], 1995). The soil was classified as loamy, with a texture of 48.8% sand, 38.6% silt, and 12.6% clay (ISO 11277; International Organization for Standardization [ISO], 2020). Soil metal concentrations were 6.2 g kg^−1^ Al, 43.2 g kg^−1^ Ca, 13.3 g kg^−1^ Fe, 15.8 g kg^−1^ Mg, and 0.446 g kg^−1^ P (Environmental Protection Agency [EPA], 2007).

Each bottle received 20 g of soil, while sterilized sand (autoclaved at 120°C for 55 min) was used for abiotic controls. Serum bottles were closed using chlorobutyl‐isoprene blend/fluorinated ethylene propylene faced septa (13 mm × 20 mm; Wheaton) before flushing with N_2_ gas (ultra‐high purity, 99.999%) to create an inert atmosphere. Each serum bottle received 1 mL of the applicable treatment solution and 1 mL of a 400 ppm benzene solution (Sigma Aldrich, 319953). Finally, abiotic controls received 2 mL of a 100 ppm stock solution of benzene.

Headspace PHC sampling in the serum bottles was performed using solid‐phase micro‐extraction (SPME) gas‐chromatography‐flame ionizing detector/mass spectrometer (Schryer & Siciliano, 2024). Briefly, benzene headspace samples were collected once a week for 5 weeks using an SPME carboxen/polydimethylsiloxane fiber (57344‐U, Millipore Sigma) fitted into a manual sampler. The SPME fiber remained in the microcosm headspace for 5 min to promote the highest possible sorption. Subsequently, the sampler was allowed to desorb for 1 min at 290°C in the sampler of the gas chromatograph (GC; Model SCION 436‐GC, Bruker). The GC was installed with an HP‐5MSUI column (Agilent Technologies). The methodology comprised starting the GC analysis at 60°C, increasing to 110°C at 15°C per min, followed by a 5°C per min increase to 120°C, before finally rising to 150°C at 60°C per min and held for 4.17 min. Before sampling the next microcosm with the fiber, the system was placed in the bakeout station at 290°C for 10 min to remove any remaining benzene. The flame ionization detector data were collected, and an external calibration curve quantified benzene concentrations. Peak areas were transferred from the CompassCDC (Scion Instruments) software to R to complete the analysis.

### 2.3 Determining the biofouling risks within the pea gravel columns

Pea gravel‐filled columns were used to review biofouling risks, where Figure S2 provides an experimental design schematic. Twelve columns were employed (as previously described in Section 2.2). Total porosity and void volume in the gravel columns were calculated using Equation (1):

(1) $$n=1-\left(\frac{\mathrm{BD}}{\mathrm{PD}}\right)$$

where *n*, BD, and PD are the porosity (%), bulk density (cm^3^), and particle density (cm^3^) of the gravel, respectively. The void volume was measured as shown in Equation (2):

(2) $$V_{V}=V\times n$$

where *V_v_* is the gravel column void volume (cm^3^), and *V* is the total gravel column volume (cm^3^).

Pea gravel columns were filled with either sterilized pea gravel (*n* = 6) or pea gravel inoculated with *Pseudomonas putida* (ATCC 17484, American Type Culture Collection [ATCC]) or with sterilized pea gravel (autoclave cycle at 121°C; *n* = 6). The columns were divided into two treatments: receiving the IRC solution (*n* = 3) or a Milli‐Q control (*n* = 3).

The methodology by Puhm et al. (2022) was used to promote biofilm formation. Briefly, lysogeny broth (LB) liquid medium (10 L^−1^ of tryptone [T9410, Sigma], 5 g L^−1^ of yeast extract [Y1625, Sigma], and 10 g L^−1^ NaCl [S9888, Sigma]) was inoculated with *Pseudomonas putida* (ATCC 17484). Approximately 30 mL of the inoculated medium was added to half‐filled 50‐mL centrifuge tubes containing the sterilized pea gravel. The tubes were incubated at 37°C on a shaker at 200 rpm for 48 h. A sterile tube containing sterile pea gravel and sterilized LB medium was included as a control.

Radioactivity measurements were performed at the Saskatchewan Centre for Cyclotron Sciences. The experiment followed the procedure described by Mamet et al. (2021) and illustrated in Figure S3. Briefly, two ^18^F‐labelled compounds were used for the experiment: [^18^Fluoride (F)] F^−^_(aq)_ and [^18^F] FDG. Approximately 76 mL (pore volume) of 220 MBq [^18^F] F^−^_(aq)_ was pumped through the columns using a peristaltic pump at a rate of 1 mL min^−1^. The column inlet and outlet tubes were plugged to maintain the solution inside the core for 90 min. Subsequently, the plug at the bottom of the column was removed (allowing the [^18^F] F^−^_(aq)_ solution to drain), and three times the pore volume (228 mL) of deionized water was pushed through the columns to remove residual [^18^F] F^−^_(aq)_. The columns were imaged for 5 min using a PET scanner (Model GNEXT PET/CT, SOFIE). After imaging, the columns rested overnight to let [^18^F] F^−^_(aq)_ decay. The next day, approximately 76 mL (pore volume) of 220 MBq [^18^F] F‐FDG was pumped through the columns using a peristaltic pump at a 1 mL min^−1^ rate. The effluent outlet tube at the bottom of the columns was plugged to maintain the solution inside for 90 min, promoting microbial uptake of the [^18^F] F‐FDG. Subsequently, the plug at the bottom of the column was removed (allowing [^18^F] F‐FDG solution to drain), and three times the pore volume in deionized water (228 mL) was pushed through the column to remove residual FDG, leaving only FDG inside bacterial cells. Finally, the columns were imaged for 5 min using a PET scanner.

Images were processed and analyzed using ImageJ software (version 1.53t; Schneider et al., 2012). The [^18^F] F^−^_(aq)_ results represent open voids unoccupied by bacteria (gravel open pore volume), while [^18^F] F‐FDG analysis quantified open voids plus bacterial volumes (gravel porosity). The difference between the FDG and F^−^ values represents pea gravel core bacterial volumes.

### 2.4 Statistical analysis

The ion concentration in the effluent from columns treated with IRC solution was compared with negative controls using one‐way analysis of variance (ANOVA). Breakthrough curves and equilibrium time in the pea gravel of each ion were isolated using effluent concentrations of the IRC solution‐treated columns. Equilibrium was achieved when the ion concentration at the inlet equalled the concentration at the outlet. The Shapiro–Wilk test, quartile‐quartile plots, and histograms were used to test normality. Depending on the number of groups to compare, either an ANOVA or the Tukey honestly significant difference test was used to determine treatment differences (*p* < 0.05). Benzene degradation differences between treatments were isolated using a linear mixed model with experimental units as random effects from repeated measurements over time (Table S1).

First‐order rate constants approximated benzene degradation rates for the predicted minimal to no microbial population growth in the soil experimental units (Aronson & Howard, 1997). The simplified form of the first‐order kinetic model is provided as Equation (3):

(3) $$\mathrm{Ln}\left(\frac{C_{0}}{C_{t}}\right)=-kt$$

where *C_0_* and *C_t_* are the initial and residual concentrations of the substrate (benzene concentration as mg L^−1^), *k* is the first‐order rate constant (days^−1^), and *t* is the exposure time (days; Iqbal et al., 2018).

## 3 RESULTS

**FIGURE 2:**
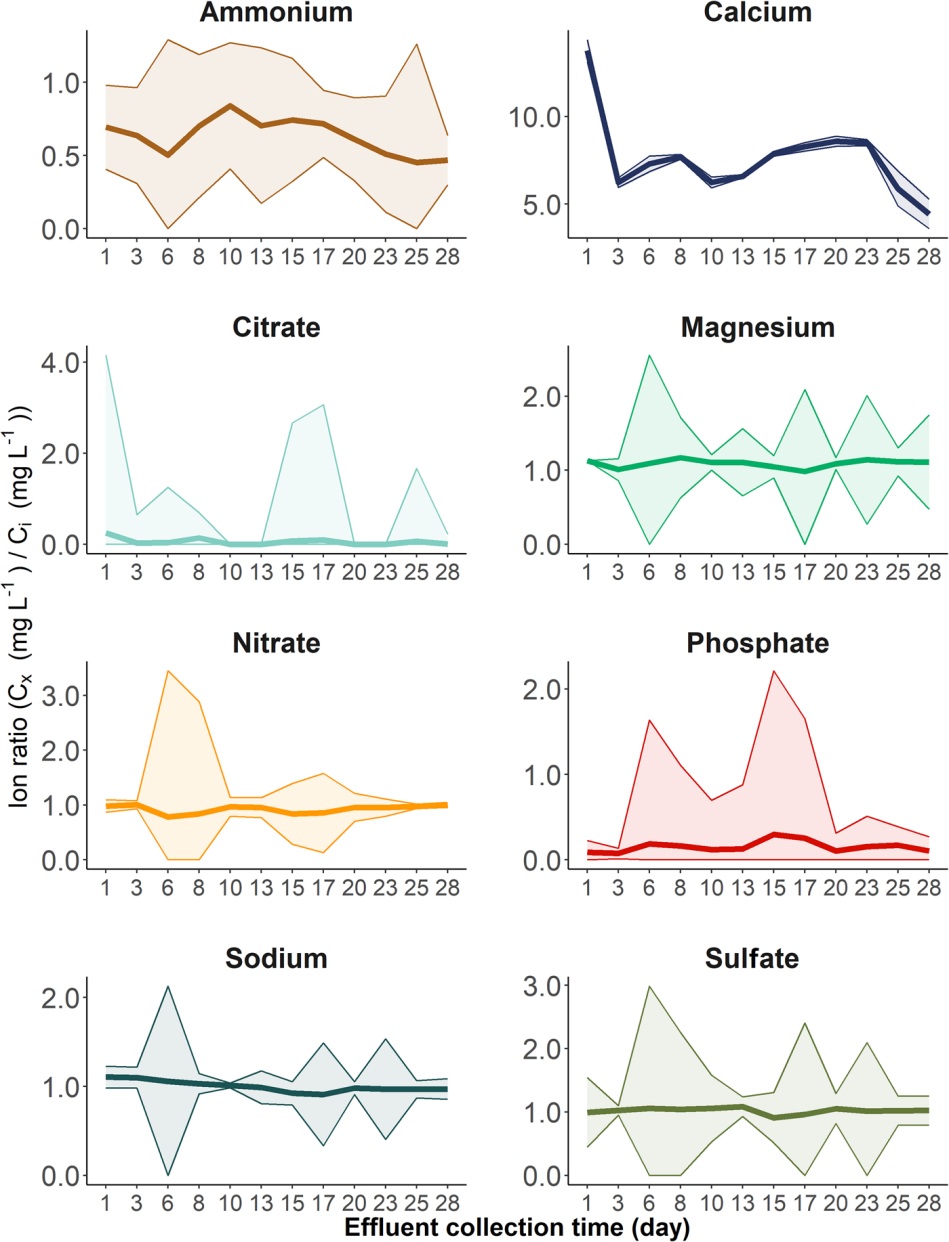
Ratio of IRC solution ion concentration (*n* = 3, mg L^−1^) ± standard error (represented by the ribbon) in the effluent to the initial ion concentration (Table 1; mg L^−1^) by incubation time after passing through the pea gravel columns. Values around 1.0 represent concentrations that mimic the initial IRC solution composition, while values below 1.0 signify ions lost via biotic or abiotic processes. Finally, values about 1.0 indicate an ion accumulation after passing the solution through the pea gravel.

The proportion of ions lost by sorption or biotic consumption varied by anion and cation type (Figure 2). Due to ratios that consistently remained close to one, Mg, NO_3_, Na and SO_4_ were the most stable ions in the initial IRC solution following pea gravel column elution. Calcium concentrations were significantly increased following elution through the pea–gravel column, starting at 13.8 from day 1 to 4.4 at 28 days. Finally, the citrate, NH_4_, and PO_4_ ratio was below 1.0, suggesting loss from processes within the pea gravel column.

**FIGURE 3:**
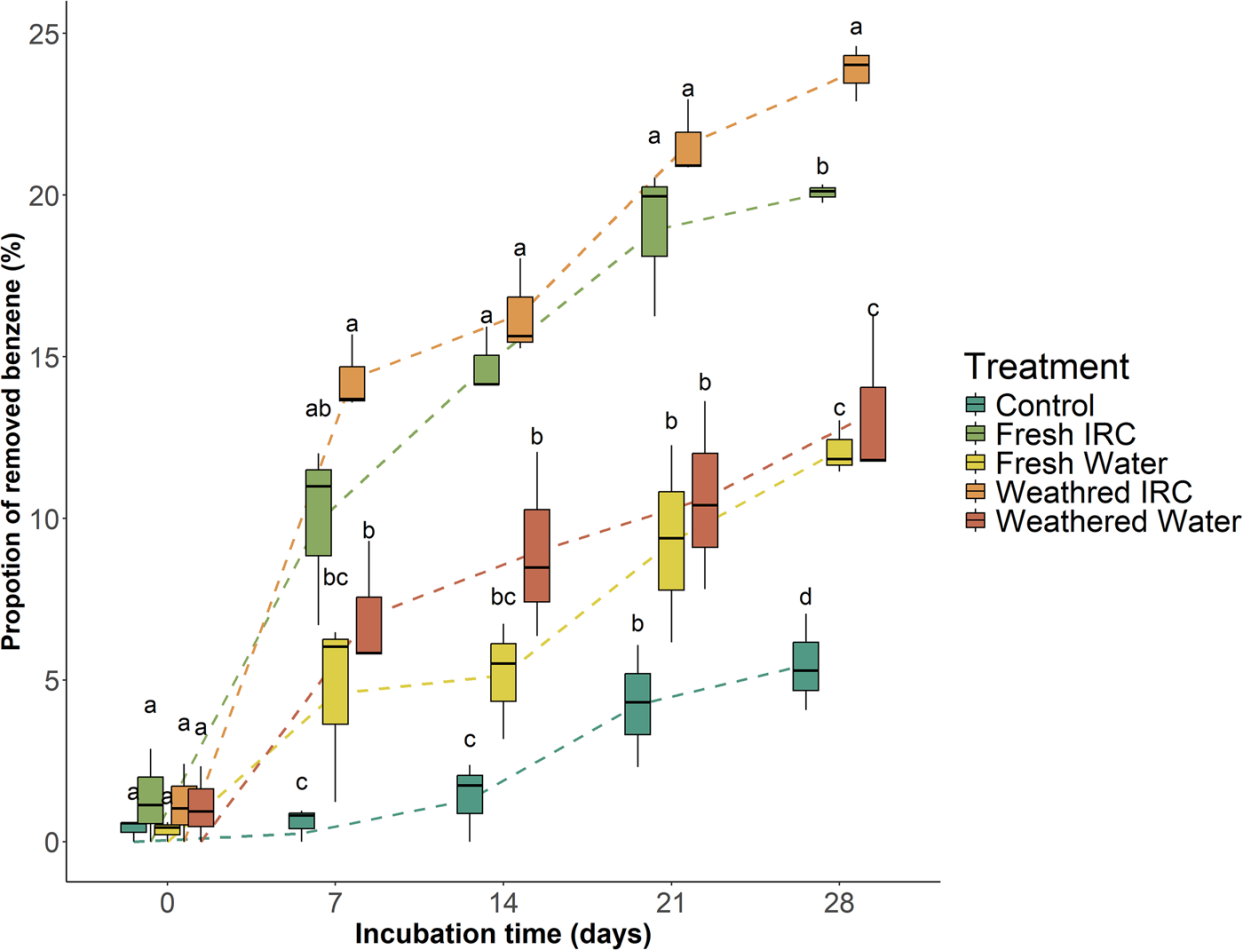
Proportion of removed benzene (percent, mean ± standard error) for a treatment across the incubation time, measured by solid‐phase micro‐extraction. The proportion of degraded benzene was calculated by dividing the measured mean of benzene (mg L^−1^) at the time of interest by the mean of time zero benzene. Letters of significance denote differences in the means identified by the Tukey honestly significant difference (HSD) test (*p* ≤ 0.05).

Passing the IRC biostimulatory solution through pea gravel columns did not inhibit benzene degradation (Figure 3). Differences between treatments become apparent after just 7 days. The weathered IRC‐doped microcosm (i.e., microcosm doped with the IRC biostimulatory solution after passing through the gravel columns) had the highest degradation (14 ± 0.7%) but was statistically similar to the fresh IRC‐doped microcosms (Table S1). After 14 and 21 days, the fresh (15 ± 0.6% and 19 ± 1.3%) and weathered IRC (16 ± 0.9% and 22 ± 0.7%) solutions promoted the greatest benzene degradation, while the fresh water was the least successful treatment (5.1 ± 1.0% and 9.3 ± 1.8%) and was comparable to the control (1.3 ± 0.8% and 4.2 ± 1.1%). Finally, benzene biodegradation in the weathered IRC (24 ± 0.5%) solution microcosms outperformed all treatments at day 28.

Effluent IRC solution outperformed all other treatments (Table 2). The first‐order degradation trends of the fresh IRC treatment (0.010 ± 0.002 days^−1^) doubled the degradation rate constant compared with the fresh water treatment (0.005 ± 0.002 days^−1^). Only fresh IRC and weathered IRC rate constants were statistically (*p* < 0.05) greater than the abiotic control. The increased degradation rates correspond to half‐life times of 345 days for the abiotic control, 87 days for the weathered IRC treatment, and 71 days for the fresh IRC treatment. Finally, no statistical difference (*p* > 0.05) was measured between the fresh IRC solution and its weathered counterpart.

**TABLE 2:** First‐order kinetic constant parameters for the anaerobic biodegradation of benzene in soil for different treatments using microcosms (*n* = 15). Degradation coefficients are presented in days ± standard deviation. Half‐life indicates the time required to decrease the initial concentration to one‐half (50%).

Treatment	K (days^−1^)	Half‐life (days)	*R* ^2^
Fresh IRC	0.010 ± 0.002a	71.3	0.96
Weathered IRC	0.008 ± 0.001ab	86.8	0.97
Fresh water	0.005 ± 0.002bc	136	0.97
Weathered water	0.005 ± 0.000c	150	0.99
Control	0.002 ± 0.000c	345	0.95

*Note*: Letters of significance denote differences in the means identified by the Tukey honestly significant difference (HSD) test (*p* ≤ 0.05).

Gravel porosity estimates using [^18^F] F^−^_(aq)_ and [^18^F] FDG radiochemistry were consistent with calculations made based on bulk density and particle density (Table 3). The particle density for the gravel was assumed to be 2.65 g cm^−3^, the total porosity of the gravel pea columns was 36%, and the total volume of the columns was 113 cm^3^. Overall, PET results were consistent with estimates based on bulk density. Columns treated with IRC showed twice the bacterial volumes than columns treated with Milli‐Q water. Bacteria occupied 8.7%–13% of the total pore volume in the columns treated with IRC solution and 3.9%–6.5% in the columns treated with water. One‐way ANOVA demonstrated that bacterial volumes were statistically different (*p* = 0.0212) in columns treated with IRC solution versus columns treated with Milli‐Q water.

**TABLE 3:** Laboratory‐ and radiochemistry‐derived void characteristics of IRC biostimulatory solution and Milli‐Q water eluding pea gravel columns.

		Laboratory				Radiochemistry			
Sample ID	Treatment	Gravel mass (g)	Gravel volume (cm^3^)	Bulk density (g cm^−3^)	Porosity (cm^3^)	Bacterial volume (cm)	Open pore volume (cm)	Porosity (cm)	Space occupied by bacteria (%)
1	IRC biostimulatory solution	187	112	1.7	40	3.7	39	42	8.8
2		186	111	1.7	40	5.7	37	43	13
3		186	111	1.7	40	4.7	37	42	11
4	Water	186	111	1.7	40	2.8	40	43	6.6
5		187	112	1.7	40	2.5	42	45	5.6
6		187	111	1.7	40	1.8	44	46	3.9

*Note*: The laboratory columns represent the calculated porosity based on gravel mass (g) and the volume of the sheathing (cm3). The radiochemistry columns represent the data collected using 2‐deoxy‐2‐[18F] fluoro‐D‐glucose positron emitting tomography, where total pores and [18F] fluoride identified open pores. Bacterial volume was calculated by subtracting the open pore volume from the total pore volume. The space occupied by bacteria (%) was calculated by dividing bacterial volume (cm3) by column porosity (cm3). Water = Milli‐Q (Millipore Sigma, Model Direct 16).

**FIGURE 4:**
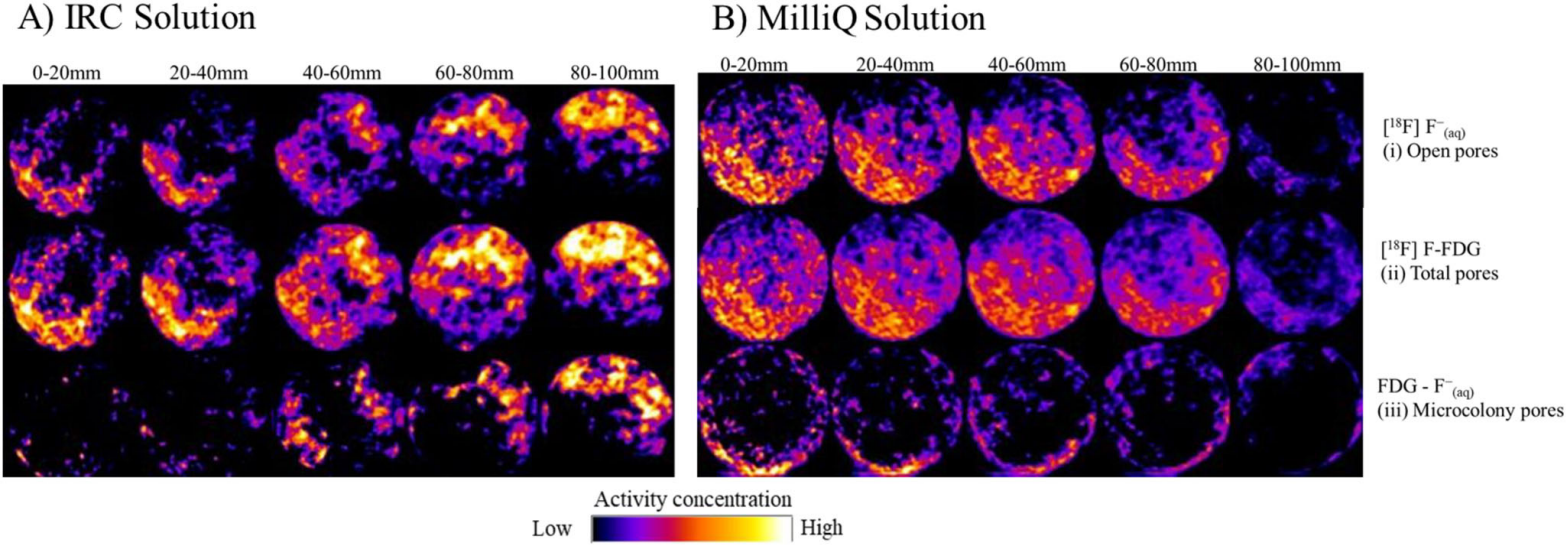
Mean radioactivity at different depths in columns packed with pea gravel and treated with (A) IRC solution and (B) Milli‐Q solution using positron emitting tomography. Radioactivity concentrations were adjusted relative to their maximum values to enhance visual comparisons between the images. Notably, the actual concentrations in the residual images were lower than the standardized maximum values. We differentiated between total pore volume as pores in which all the [^18^F]‐2‐deoxy‐2‐[^18^F]fluoro‐D‐glucose (FDG) was present (ii) and open pores in which only [^18^F]Fluoride (F)^−^_(aq)_ was present (i). As [^18^F]F^−^_(aq)_ is not actively taken up by microorganisms but [^18^F]FDG is, we can calculate the total bacterial volume as [^18^F]FDG–[^18^F]F^−^_(aq)_. We differentiated soil pores as those in which bacteria live within the pore volume, that is, microcolony pores, and soil pores in which bacteria only live on the walls, presumably in a surface biofilm (iii).

Radioactivity in pea gravel columns treated with the biostimulatory solution was greater than in Milli‐Q‐treated columns (Figure 4). For the IRC‐treated pea gravel, the area representing open pores (estimated using [^18^F]–^−^F_[aq]_) increased at increasing depths down the pea gravel column (top row, Figure 4A). The first two slices (i.e., 0–20 mm and 20–40 mm) of the total pore measurement (isolated using [^18^F]–FDG) possessed more black space (middle row, Figure 4A). Finally, the difference between the values identified more of the microcolony pores at the bottom of the pea gravel core (bottom row, Figure 4A). For the pea gravel core treated with the Milli‐Q eluent, the top of the column possessed greater open pores (estimated using [^18^F]–^−^F_[aq]_) than the bottom (top row, Figure 4B). Unlike the IRC‐treated pea gravel column, there are fewer total pores (isolated using [^18^F]–FDG) in the top of the column (0–80 mm) than in the bottom section (80–100 mm; middle row, Figure 4B). Finally, the bacterial community in the Milli‐Q is on the edges of the sheathing rather than the bottom of the pea gravel core (bottom row, Figure 4B).

**FIGURE 5:**
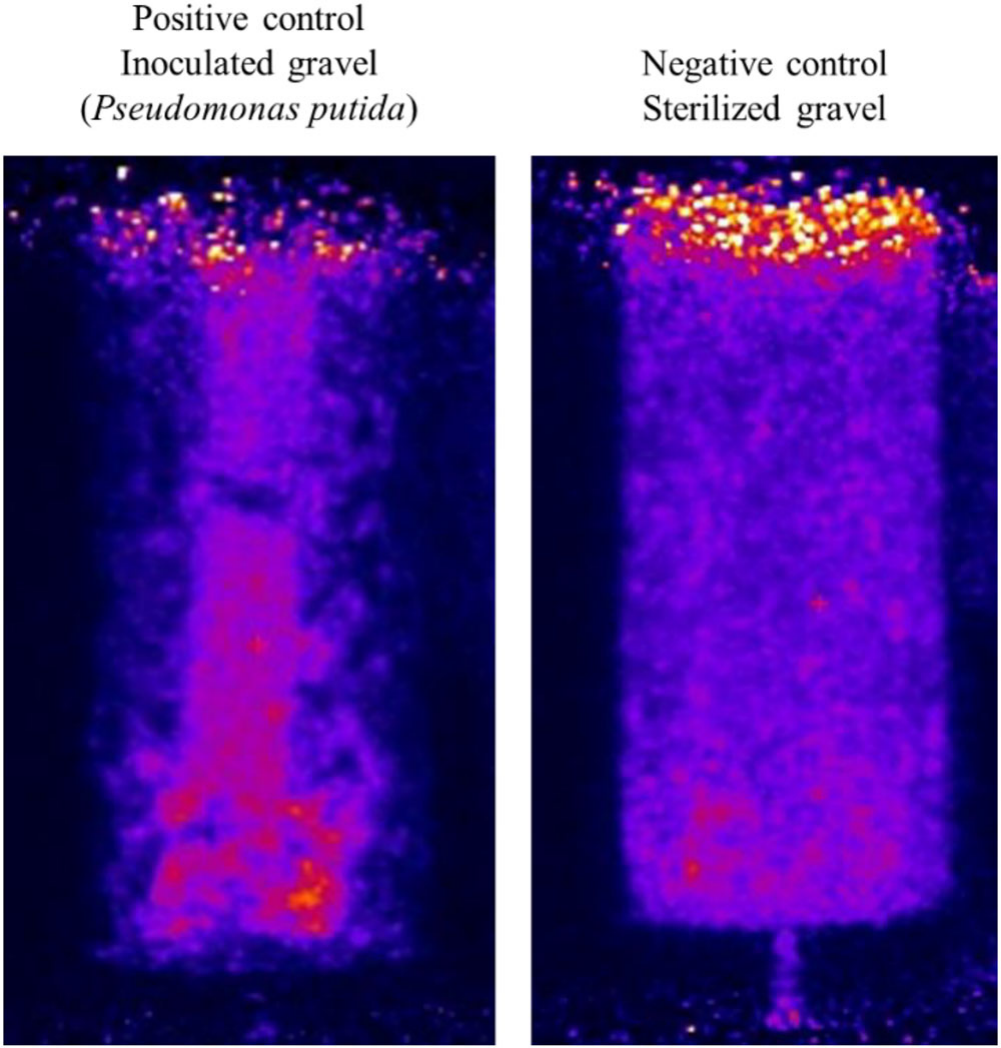
Mean radioactivity in positive and negative controls dosed with [^18^F]‐2‐deoxy‐2‐[^18^F]fluoro‐D‐glucose using positron emitting tomography. Reduced exposure in the positive control allows for enhanced visualization of active bacteria in the center of the column.

Adding the IRC solution promoted greater microbial activity than in the sterilized pea gravel column (Figure 5). For the IRC solution‐treated columns, metabolically active *Pseudomonas putida* occupied large proportions of the total pore space (Figure 5A). In contrast, the negative control (sterilized gravel) exhibits only open pores (Figure 5B).

## 4 DISCUSSION

Using pea gravel columns to mimic UGST tank beds provided new insights into how microbiology and mineralogy could influence PHC bioremediation success. First, mostly biological and, to a much lesser extent, abiotic processes cause ionic compositional changes as the biostimulatory solution passes through the coarse particles. Open pores suggest that eluting the IRC solution through pea gravel does not promote biofilm formation, while the ensuing effluent promotes greater PHC degradation in anaerobic systems. Consequently, passing the solution through the same UGST pea gravel channels that direct the flow of PHC to neighboring finer particle soils may increase the bioremediation success rates.

### 4.1 Compositional changes to the IRC biostimulatory solution following elution

Passing the IRC biostimulatory solution through pea gravel altered the effluent composition by desorbing considerable Ca. Unlike the other tested ions, the ratio of dissolved Ca in the effluent to the initial biostimulatory solution increased dramatically (from 14 on day 1 to 4.4 on day 28), largely absent from the bead and resin controls (Figures S4 and S5). As gravel typically contains traces of Na, Ca, and Mg (Zhang & Luo, 2019), pea gravel likely leached significant amounts of Ca into the solution.

Ammonium and citrate concentrations in the pea gravel effluent were lower than in the initial biostimulatory solution. Presumably, rapid immobilization of the available dissolved components occurred as the IRC biostimulatory passed microorganisms surviving in limited N and C conditions (Pang et al., 2022; Ström et al., 2005; Wang et al., 2023). Generally, the IRC biostimulatory solution supported microbial proliferation, as the proportion of bacteria occupying space in the IRC‐treated pea gravel cores (8.7%–13%) was greater than in the water‐treated pea gravel columns (3.9%–6.5%). However, unlike the NH_4_, effluent citrate concentrations were negligible in pea gravel (Figure 2) and reduced over time in the glass bead columns (Figure S4). The citrate ions may have precipitated out of the solution by forming insoluble organo‐metallic complexes with target metals (e.g., Ca; Ström et al., 2005) and were filtered out before IC analysis.

TPP degradation processes caused phosphate concentration variability in pea gravel effluent over time. TPP hydrolysis can occur spontaneously in aqueous solutions at room temperature (Torres et al., 2005) or via the breakdown of TPP through phosphatase activity (Hamilton et al., 2017). Abiotic pea gravel TPP hydrolysis rates were negligible, as phosphate concentrations were small in the control glass bead column (Figure S4). Likely, the dissolved phosphate concentration peaks at days 6 and 15, representing increased rates of enzymatic TPP degradation. Phosphatase activity relates to microbial community composition, temperature, and ecological succession stage, among other factors (Margalef et al., 2017). As ambient temperature was one of the few factors not controlled during the trial, the capacity of the enzyme to release phosphate from TPP may have increased during those brief periods. However, as most UGST pea gravel tanks are cooler than ambient air, TPP loss to enzymatic hydrolysis is assumed to be negligible (Hamilton et al., 2018).

Finally, stable nitrate and sulfate concentrations in the pea gravel column effluent will promote PHC degradation in the anaerobic neighboring finer particle soil. As we did not control fluid oxygen concentrations, the anaerobic electron acceptors were not required for proliferation (Liu et al., 2020). Depending on groundwater depth and its subsequent influence on soil moisture content, the larger pore geometry will allow deeper oxygen penetration before complete consumption (Du et al., 2023). Consequently, a greater proportion of the electron acceptors may be introduced to the microorganisms responsible for anaerobic PHC degradation (He et al., 2022).

### 4.2 Microbial growth in the pea gravel column

While microorganisms did not grow on the walls of the IRC solution‐treated pea gravel columns, biota did accumulate at the bottom (80–100 cm; Figure 4). Wall flow (or sidewall flow) commonly occurs in soil column studies. Fluids will leak into gaps between the column and the sheathing, creating an unrepresentative preferential flow pathway (Lewis & Sjöstrom, 2010; Richter, 2002). This phenomenon is apparent in the microcolony pores of the Milli‐Q‐treated cores (Figure 4B). In comparison, as the locational difference in [^18^F]‐FDG activity between layers of the IRC solution‐treated pea gravel is dissimilar, preferential pathways do not appear as the media moves through the column (Figure 4A). In the IRC‐treated *Pseudomonas putida* inoculated core, the microbes preferentially grow near the center of the core, with a larger proportion near the bottom of the core (Figure 5). Yet, scans at the bottom of the core (Figures 4 and 5) suggest that gravitational forces caused IRC solution nutrients to accumulate before discharging from the system. As nutrient availability is the limiting factor for microbial growth, the trapped precipitates promoted biotic growth with all three pore types. Finally, the surface of the sterilized gravel core reported increased microbial radioactivity, likely due to the exposure of the core to a non‐sterilized environment during sampling (Figure 5).

Pea gravel promoted less microbial propagation than similar conditions identified within mineral soil. For example, Mamet et al. (2021) used ^18^F‐FDG to show that bacteria occupied between 60% and 74% of the total pore space in soil columns treated with biostimulatory solutions of varying composition. The bacteria in our pea gravel cores occupied a much smaller fraction (8.7%–13%). Generally, biofilms form as particle nutrients and organisms accumulate on the surface of highly porous matrices, including gravel (Tianzhi et al., 2014). As gravel has a smaller surface area than soil, there is a reduced risk of biofilm formation that causes clogs in preferential flow paths (Aufrecht et al., 2019; Mamet et al., 2021).

### 4.3 Effluent potency in supporting PHC biodegradation

Soils that received IRC biostimulatory solution increased anaerobic soil benzene biodegradation rates over Milli‐Q and control treatments. While the benzene first‐order degradation rates (0.0015–0.522 days^−1^; Table 3) fall within ranges found in the literature (Aronson & Howard, 1997; Suarez & Rifai, 1999), the soils receiving IRC solution effluent outperformed the Milli‐Q effluent soils. As many soils in Saskatchewan are nutrient‐limited for PHC biodegradation (e.g., Chen et al., 2017; Siciliano et al., 2016b), the additional nutrients stimulated the present microbial communities to degrade benzene at greater rates (Karppinen et al., 2017). As the degradation rates of PHC are often lower than the ambient temperatures used during the trial (Ferguson et al., 2003), the reported half‐lives suggest natural attenuation rates would be negligible at this site.

Surprisingly, relative to the fresh IRC solution, the weathered IRC solution promoted greater benzene removal after 28 days in the anaerobic soil microcosms. Previous studies have shown that microbial communities can be transported down the soil profile, altering the microbial subsurface soil community composition (Gammack et al., 2021). Consequently, the pea gravel may act as a conductor of active microorganisms by introducing bioaugmentation (Omenna et al., 2024) during nutrient addition.

## 5 CONCLUSION

While manufactured preferential pathways promote contaminant migration, employing the same pathways to distribute biostimulatory solution will improve PHC bioremediation rates. Results show that changes to the biostimulatory solution following elution increased PHC degradation within soil microcosms. Due to lower proportions of active microbial communities, fewer resources were consumed in the pea gravel columns compared to soil studies. Certain key nutrients (i.e., nitrate and sulfate) were largely unaffected by pea gravel, theoretically increasing transport ranges. Biofilm formation risks are minimal, as microorganisms in the pea gravel core after IRC solution elution largely proliferated due to nutrient accumulation from gravitational forces. Finally, adding IRC effluent to the mineral soil microcosms increased benzene degradation compared to fresh IRC solution due to an enhanced microbial presence.

However, whether changes found in the biostimulatory solution composition will negatively influence long‐term PHC biodegradation in UGST tank nests remains unclear. For example, as the pea gravel leaches high amounts of Ca, citrate and PO_4_ may precipitate and become biologically unavailable. While our laboratory‐based results confirm that the material is suitable for transporting biostimulatory solution, further investigations are required to ensure results may be extrapolated to on‐site scenarios.

## AUTHOR CONTRIBUTIONS

**Aimée D. Schryer**: Data curation; formal analysis; methodology; writing—original draft. **Alejandro Alvarez Ruiz**: Conceptualization; data curation; formal analysis; investigation; methodology; writing—review and editing. **Kris Bradshaw**: Conceptualization; project administration; writing—review and editing. **Steven D. Siciliano**: Conceptualization; funding acquisition; project administration; supervision; writing—review and editing.

## CONFLICT OF INTEREST STATEMENT

The authors declare no conflicts of interest.

## Supporting information




**Figure S1**: Schematic of the experiment set‐up (section 2.2 of the main manuscript) used to compare the concentration of ions following elution through the column of interest.
**Figure S2**: Schematic of the experiment set‐up (section 2.3 of the main manuscript) used to determine benzene degradation within soil experimental units.
**Figure S3**: Schematic of the radioactive tracer experimental set‐up (section 2.3 of the main manuscript) used to determine the benzene degradation within soil experimental units.
**Figure S4**: Ratio of IRC solution ion concentration (n = 1, mg L^−1^) of the effluent to the initial ion concentration (Table 1; mg L^−1^) by incubation time in the bead control columns. Values around 1.0 represent concentrations that mimic the initial IRC solution composition, while values below 1.0 signify ions lost via biotic or abiotic processes. Finally, values about 1.0 indicate an ionic accumulation after passing the solution through the beads.
**Figure S5**: Ratio of IRC solution ion concentration (n = 1, mg L^−1^) of the effluent to the initial ion concentration (Table 1; mg L^−1^) by incubation time after passing through the resin control columns. Values around 1.0 represent concentrations that mimic the initial IRC solution composition, while values below 1.0 signify ions lost via biotic or abiotic processes. Finally, values about 1.0 indicate an ionic accumulation after passing the solution through the resin beads.
**Table S1**: P‐values from the Tukey HSD pairwise multiple comparisons posthoc test to compare treatments across the entire incubation period of the benzene biodegradation experiment.
