# Supplementary material for: Analysis into the viability of pea gravel as a diffusing material for biostimulation systems in petroleum hydrocarbon‐contaminated soils
Source: J Environ Qual. 2025 Dec 17;55(1):e70114. doi: 10.1002/jeq2.70114 (PMC12710573; doi:10.1002/jeq2.70114)
Supplement: Supplementary file 1 — Figure S1: Schematic of the experiment set‐up (section 2.2 of the main manuscript) used to compare the concentration of ions following elution through the column of interest. Figure S2: Schematic of the experiment set‐up (section 2.3 of the main manuscript) used to determine benzene degradation within soil experimental units. Figure S3: Schematic of the radioactive tracer experimental set‐up (section 2.3 of the main manuscript) used to determine the benzene degradation within soil experimental units. Figure S4: Ratio of IRC solution ion concentration (n = 1, mg L−1) of the effluent to the initial ion concentration (Table 1; mg L−1) by incubation time in the bead control columns. Values around 1.0 represent concentrations that mimic the initial IRC solution composition, while values below 1.0 signify ions lost via biotic or abiotic processes. Finally, values about 1.0 indicate an ionic accumulation after passing the solution through the beads. Figure S5: Ratio of IRC solution ion concentration (n = 1, mg L−1) of the effluent to the initial ion concentration (Table 1; mg L−1) by incubation time after passing through the resin control columns. Values around 1.0 represent concentrations that mimic the initial IRC solution composition, while values below 1.0 signify ions lost via biotic or abiotic processes. Finally, values about 1.0 indicate an ionic accumulation after passing the solution through the resin beads. Table S1: P‐values from the Tukey HSD pairwise multiple comparisons posthoc test to compare treatments across the entire incubation period of the benzene biodegradation experiment. [file JEQ2-55-0-s001.docx]

SUPPLEMENTARY MATERIALS


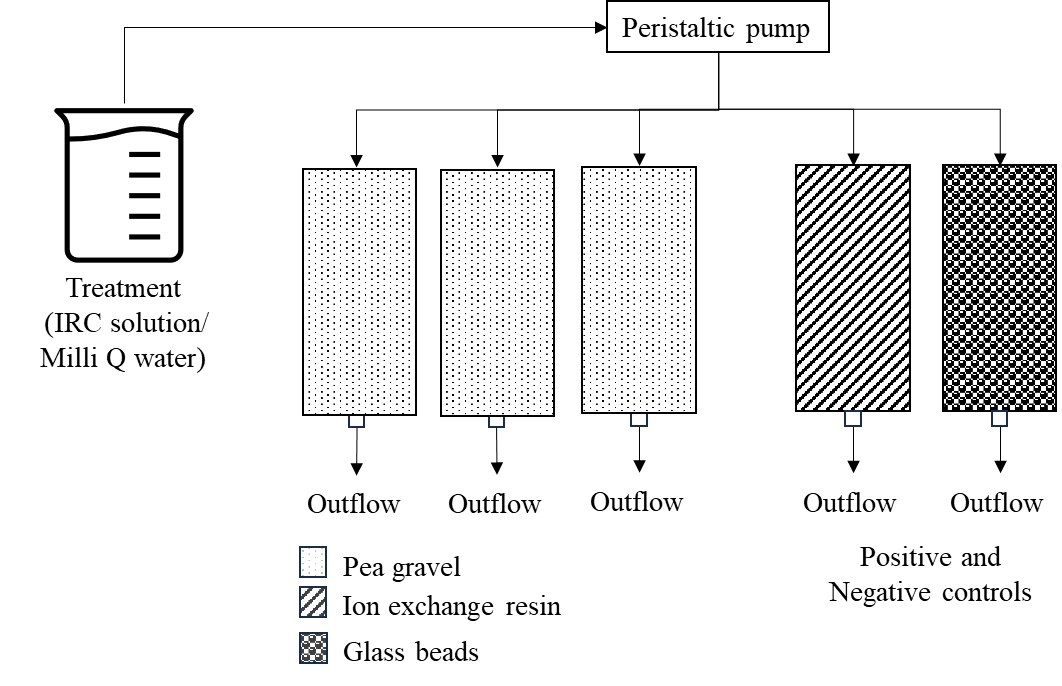


**Figure S1:** Schematic of the experiment set-up (section 2.2 of the main manuscript) used to compare the concentration of ions following elution through the column of interest.


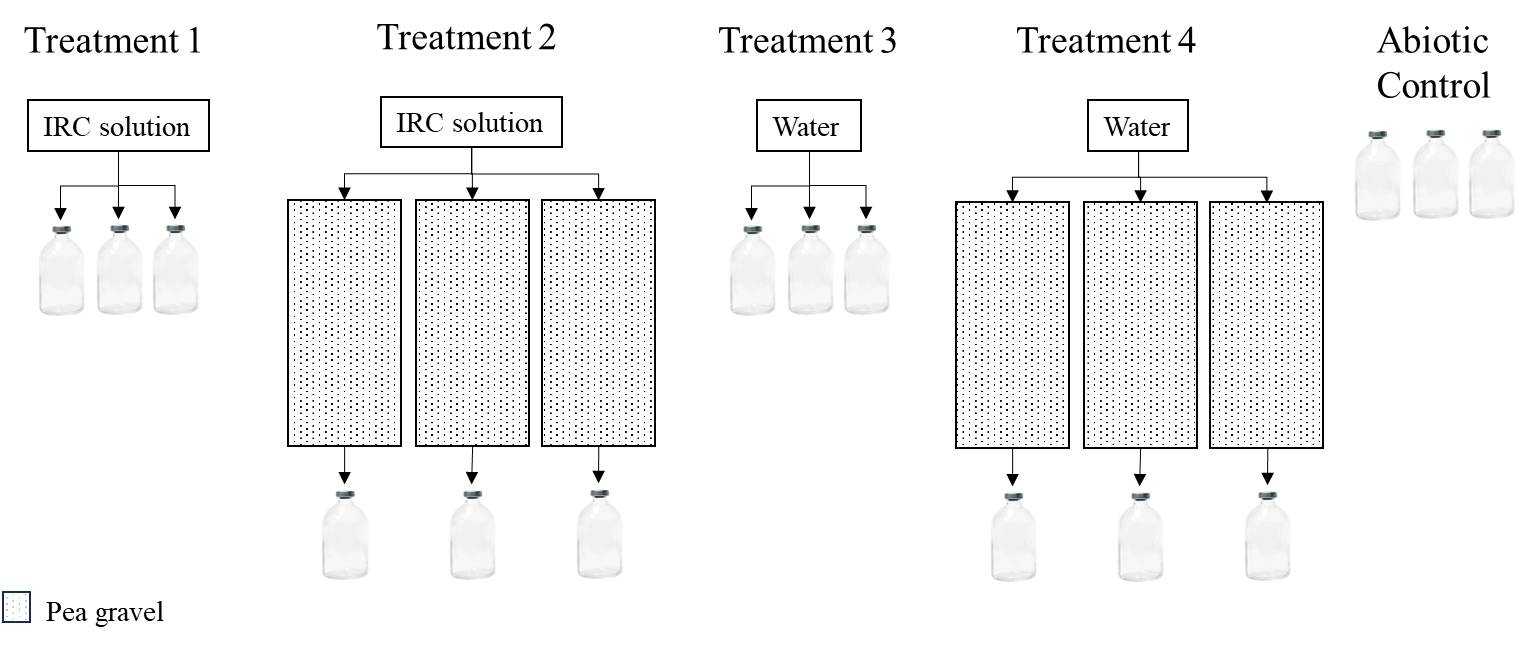


**Figure S2:** Schematic of the experiment set-up (section 2.3 of the main manuscript) used to determine benzene degradation within soil experimental units.


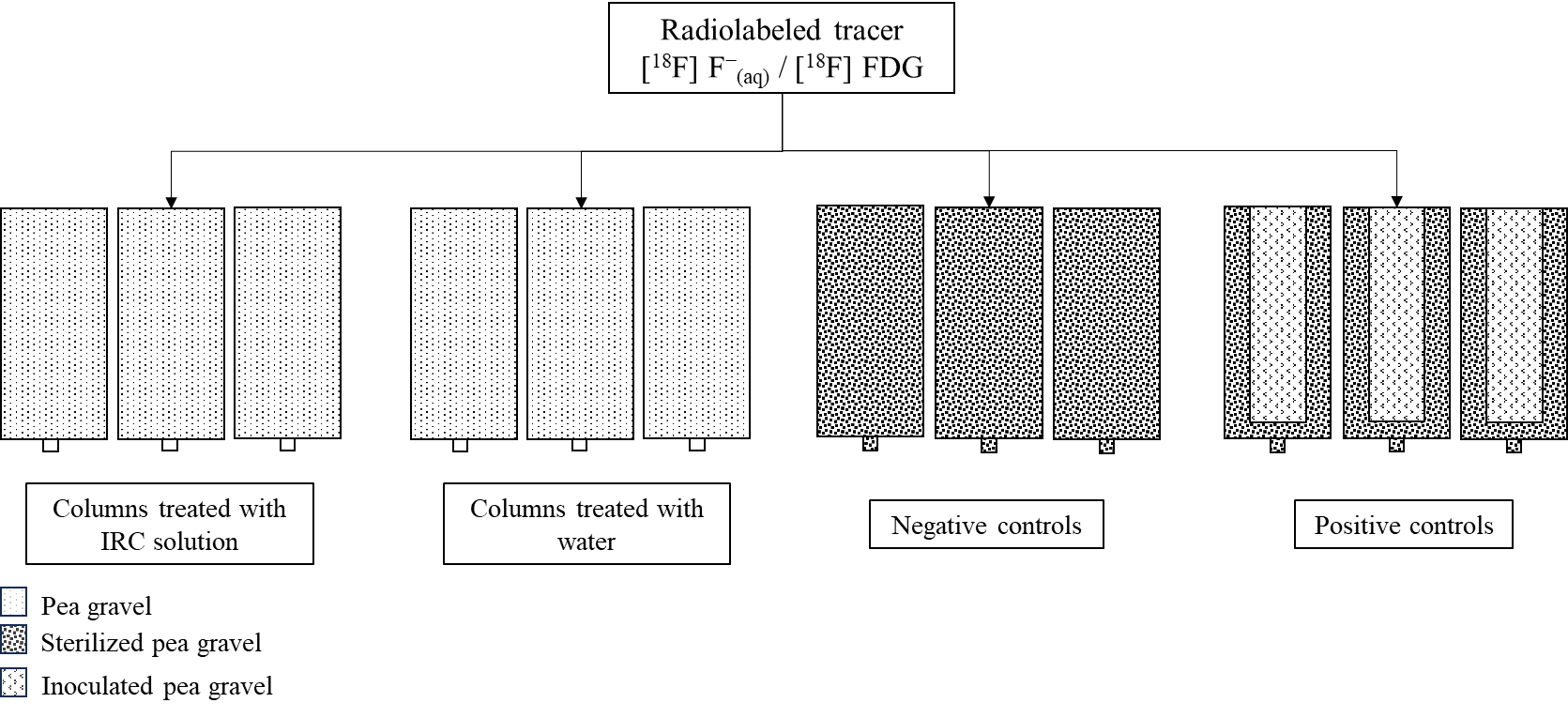


**Figure S3:** Schematic of the radioactive tracer experimental set-up (section 2.3 of the main manuscript) used to determine the benzene degradation within soil experimental units.

**Table S1:** P-values from the Tukey HSD pairwise multiple comparisons posthoc test to compare treatments across the entire incubation period of the benzene biodegradation experiment.

| Treatment | | | | |
| --- | --- | --- | --- | --- |
|  | Fresh IRC | Fresh water | Weathered IRC | Weathered water |
| Control | 0.000 | 0.017 | 0.000 | 0.000 |
| Fresh IRC |  | 0.000 | 0.275 | 0.002 |
| Fresh water |  |  | 0.000 | 0.624 |
| Weathered IRC |  |  |  | 0.000 |


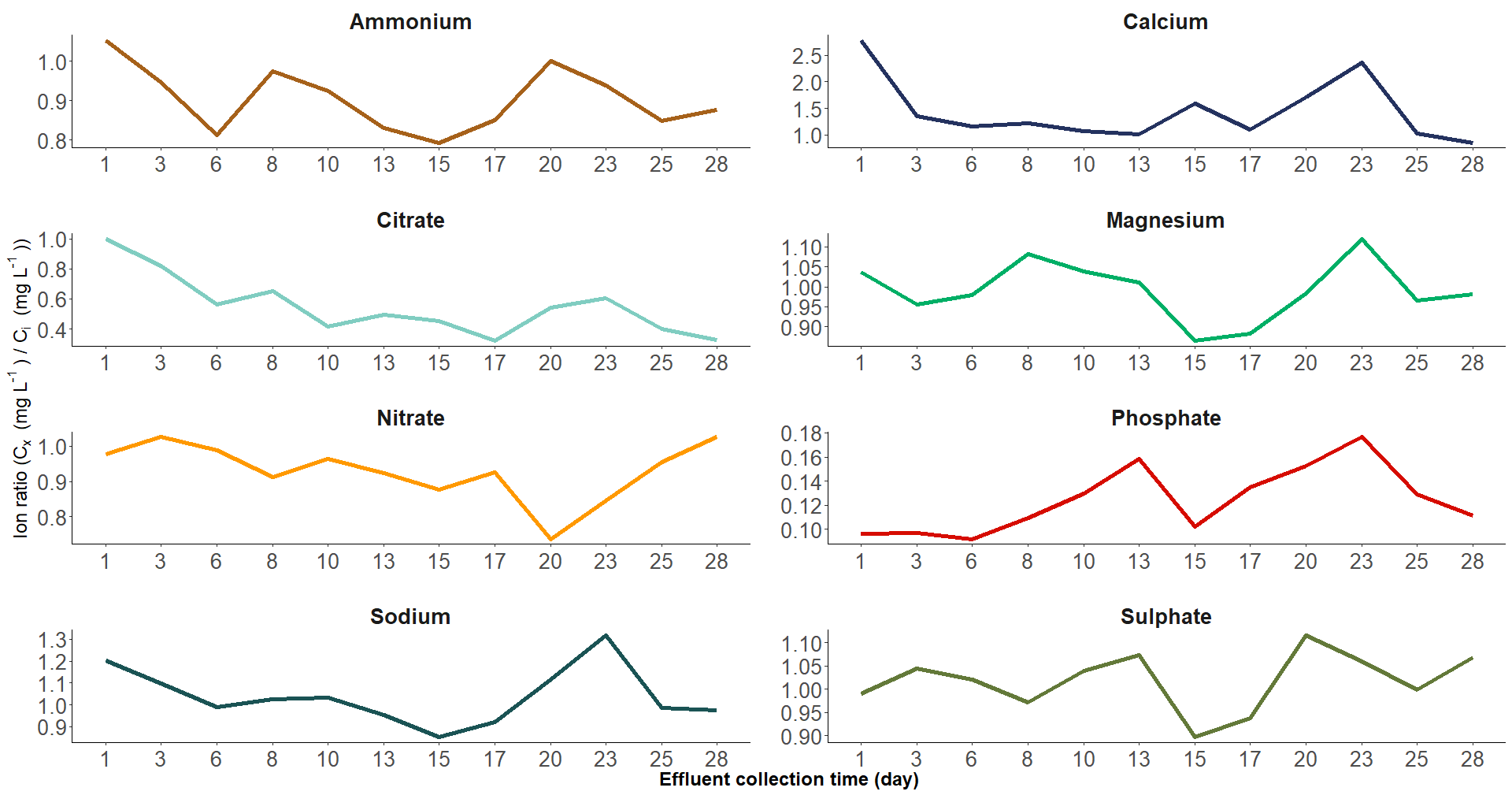


**Figure S4:** Ratio of IRC solution ion concentration (n = 1, mg L^-1^) of the effluent to the initial ion concentration (Table 1; mg L^-1^) by incubation time in the bead control columns. Values around 1.0 represent concentrations that mimic the initial IRC solution composition, while values below 1.0 signify ions lost via biotic or abiotic processes. Finally, values about 1.0 indicate an ionic accumulation after passing the solution through the beads.


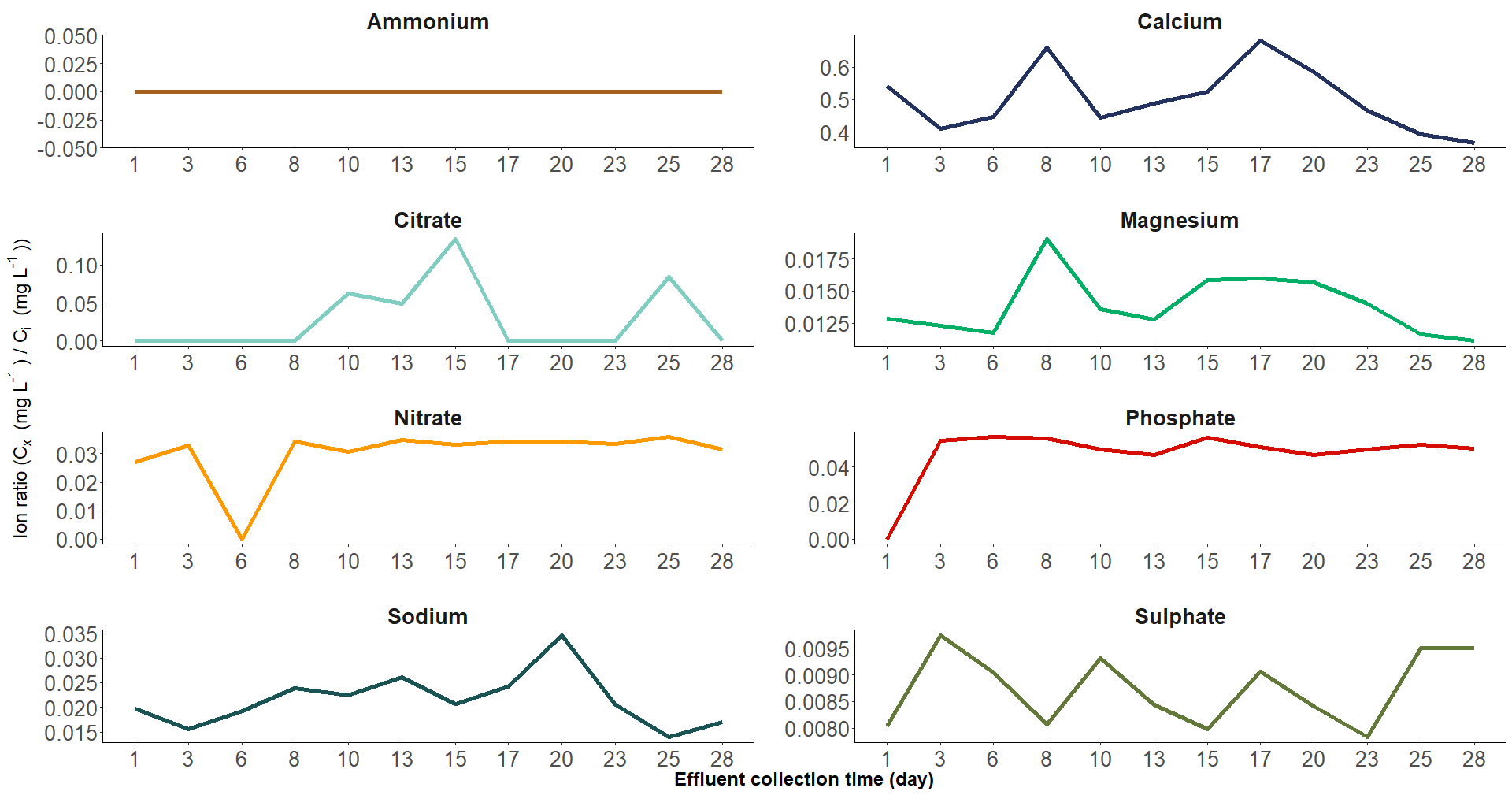


**Figure S5:** Ratio of IRC solution ion concentration (n = 1, mg L^-1^) of the effluent to the initial ion concentration (Table 1; mg L^-1^) by incubation time after passing through the resin control columns. Values around 1.0 represent concentrations that mimic the initial IRC solution composition, while values below 1.0 signify ions lost via biotic or abiotic processes. Finally, values about 1.0 indicate an ionic accumulation after passing the solution through the resin beads.
